# Transient Elastography Is the Best-Performing Non-Invasive Test of Liver Fibrosis in Obese Asian Patients with Non-Alcoholic Fatty Liver Disease: A Pilot, Cross-Sectional Study

**DOI:** 10.3390/medicina60010169

**Published:** 2024-01-17

**Authors:** Kaliyaperumal Kalaiyarasi, Acharyya Sanchalika, Low Hsien Min, Yap Wei Ming, Shelat Vishalkumar, Yew Kuo Chao, Low Jee Keem, Junnarkar Sameer, Huey Cheong Wei Terence, Tan Yen Ping

**Affiliations:** 1 Division of Hepatology and Gastroenterology, Tan Tock Seng Hospital, 11 Jln Tan Tock Seng, Singapore 308433, Singapore; kuo_chao_yew@ttsh.com.sg; 2 Clinical Research & Innovation Office, Tan Tock Seng Hospital, 11 Jln Tan Tock Seng, Singapore 308433, Singapore; sanchalika_acharyya@ttsh.com.sg; 3 Division of Radiology, Tan Tock Seng Hospital, 11 Jln Tan Tock Seng, Singapore 308433, Singapore; hsien_min_low@ttsh.com.sg; 4 Division of Pathology, Tan Tock Seng Hospital, 11 Jln Tan Tock Seng, Singapore 308433, Singapore; wei_ming_yap@ttsh.com.sg; 5 Division of General Surgery, Tan Tock Seng Hospital, 11 Jln Tan Tock Seng, Singapore 308433, Singapore; jee_keem_low@ttsh.com.sg (L.J.K.); sp_junnarkar@ttsh.com.sg (J.S.); huey_bing@ttsh.com.sg (H.C.W.T.); yen_pin_tan@ttsh.com.sg (T.Y.P.); 6 Lee Kong Chian School of Medicine, Nanyang Technological University, Singapore 308232, Singapore

**Keywords:** transient elastography, non-alcoholic fatty liver disease, liver fibrosis, non-invasive assessment

## Abstract

*Background and Objectives*: The prevalence of NAFLD (non-alcoholic fatty liver disease) is increasing, and up to 64% of Asian patients with NAFLD are obese. Non-invasive tests (NITs) for the assessment of liver fibrosis are increasingly being used, but data on their performance in obese Asian patients are lacking. In this pilot cross-sectional study, we aim to compare the distribution of serum and radiological markers of fibrosis between obese Asian biopsy-proven NAFLD patients with and without fibrosis and estimate the diagnostic accuracies of these indices. *Materials and Methods*: Obese Asian patients with NAFLD and who had undergone a liver biopsy showing histological evidence of NAFLD were invited to participate. Liver fibrosis was assessed using laboratory (APRI, AAR, BARD, FIB4, NFS, and Asia–Pacific NAFLD advanced fibrosis score) and imaging modalities (TE: transient elastography, MRE: magnetic resonance elastography, and SWU: shear wave ultrasonography). *Results*: A total of 16 patients were included in the final analysis. On liver biopsy, nine patients (56.3%) had significant fibrosis (F2 or higher), and six of these patients had advanced fibrosis (F3 or higher). F4 fibrosis was present in one patient (6.3%). For the laboratory markers, we found that the BARD score correctly identified five out of six patients with advanced fibrosis (83.4%, *p* value 0.045). Among all the NITs studied, liver stiffness measured by TE had the highest accuracy of 87.5% in its established threshold of 8.5 kPa for the detection of advanced fibrosis. MRE also performed well (81.2% in 3.64 kPa). *Conclusions*: In conclusion, TE has performed well in the detection of advanced fibrosis in obese Asian patients with biopsy-proven NAFLD in our pilot study. Further large-scale definitive studies are needed to validate the results of our findings.

## 1. Introduction

Non-alcoholic fatty liver disease (NAFLD) is defined as the excessive accumulation of triglycerides in the liver in individuals with little or no alcohol consumption. Previously thought to be a Western disease, it now increasing in prevalence among Asian countries, affecting up to 30–45% of Asian patients [1,2,3,4,5]. The presence of fibrosis remains the most important determinant of prognosis, as patients with advanced fibrosis are at an increased risk of both liver-related and cardiovascular mortality [6,7].

Obesity is a strong risk factor for NAFLD. The prevalence of NAFLD is approximately 82–98% in obese patients [4,8,9]. The quantity of liver fat, which is greater in obese patients, has prognostic implications for fibrosis acceleration [10]. One study found that the prevalence of advanced fibrosis was 17% in obese Asian patients (BMI ≥ 25 kg/m^2^, <30 kg/m^2^), and more than 50% of these patients had NASH [11]. Currently, transient elastography (TE) using the XL probe and shear wave ultrasonography (SWU) have high diagnostic accuracies in predicting liver fibrosis in obese patients [12,13,14,15,16,17,18]. However, these non-invasive tests (NITs) have their limitations, and high BMI negatively impacts the diagnostic reliability of TE in NAFLD patients, especially if the M probe is used [14]. The accuracy of liver stiffness measurements (LSMs) is operator-dependent and affected by the degree of necroinflammation in the liver, cholestasis, and hepatic congestion [19]. One study showed that even with the TE-XL probe, a reliable liver stiffness measurement could only be obtained in up to 85% of the study population [12].

Furthermore, most previous studies were undertaken in a mainly Western population. One study by Wong et al. [13] included Asians, though they made up only 30% of the study population. A Thai study [20], evaluating the diagnostic performance of NITs, found that they exhibited high diagnostic accuracies in diagnosing different stages of fibrosis in obese NAFLD and chronic hepatitis C patients. However, the limitation of this study was that the performance of these NITs was compared using magnetic resonance elastography (MRE) as a reference standard instead of liver biopsy. 

Also, the performance of magnetic resonance elastography (MRE), currently the most accurate non-invasive marker of fibrosis in NAFLD in general, has not been well assessed in obese Asian NAFLD patients [21]. 

Though liver biopsy remains the international gold standard for the detection of liver fibrosis, especially in the setting of clinical trials, it has its limitations and procedure-related complications [10,22]. There is a need to identify an accurately performing NIT for the assessment of liver fibrosis, as well as test the accuracy of the available NITs, established in Western populations, in this subset of obese Asian patients with NAFLD.

Given the paucity of data evaluating the performance of these non-invasive tests in obese Asian NAFLD patients, especially imaging-based modalities such as TE, SWU, and MRE, we aim to compare the distribution of serum biomarkers and radiological indices between obese Asian biopsy-proven NAFLD patients with and without fibrosis and estimate the diagnostic accuracies of these tests.

## 2. Materials and Methods

### 2.1. Study Design

This is a pilot cross-sectional study conducted in a single institution. Over a 10-month recruitment period, from November 2020 to June 2021, adult (between 18 and 80 years of age) obese Asian patients, defined as BMI (body mass index) ≥ 25 kg/m^2^ as per the Asia–Pacific guidelines for obesity classification in Asians, with NAFLD and who had undergone a liver biopsy showing histological evidence of NAFLD in the 6 months prior to recruitment, were invited to participate in this study.

Diagnosis of NAFLD was established via the presence of radiological evidence of steatosis, absence of regular, excessive alcohol consumption defined as >21 units (168 g) of alcohol/week for males and >14 units (112 g) of alcohol/week for females), and absence of other causes of chronic liver disease as established by negative biochemical and serological studies performed at the baseline evaluation.

Other exclusion criteria included (i) pregnancy; (ii) presence of contraindications to liver biopsy, such as coagulopathy; (iii) active or present diagnosis of hepatocellular carcinoma or other malignancies; and (iv) weight >120 kg (weight limit for magnetic resonance elastography).

Appropriate approval from the institutional domain specific review board (National Healthcare Group DSRB; Approval Code 2019/00264) was obtained before recruitment, and all patients provided their written informed consent. The study protocol was in compliance with the ethical principles of the Helsinki Declaration and followed the Good Clinical Practice guidelines.

### 2.2. Assessment of Fibrosis and Steatosis

TE was performed using Fibroscan^®^ 520 Touch (Echosens, Paris, France) on the right liver lobe using a 3.5 MHz M or 2.5 MHz XL probe according to the manufacturer’s instructions. The M probe was used initially, followed by the XL probe if the M probe failed. The TE result was considered valid if there were more than 10 successful measurements and an interquartile range (IQR)/median ratio < 30%. The median of 10 successful readings expressed as kilopascals was used as the final result for the analysis [23,24].

**Figure 1:**
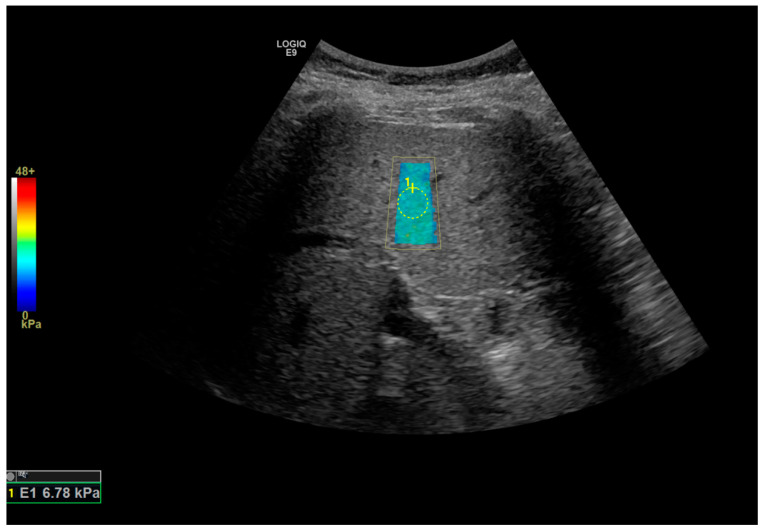
Image from the shear wave elastography study showing the circular region of interest being placed over the liver parenchyma in the right liver lobe, taking care to avoid large vessels.

**Figure 2:**
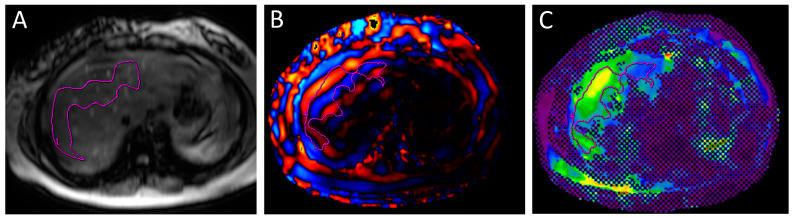
Images from an MRI elastography image showing the (**A**) magnitude images; (**B**) wave images; and (**C**) elastography map. A region of interest (ROI) was manually drawn on the elastogram image over the right lobe of the liver (purple), taking care to avoid the hexed-out areas. The ROI was propagated onto the wave, and magnitude image and adjustments were made to avoid large vessels.

The 2D-SWU examinations were performed by a sonographer with more than 20 years of experience under the supervision of a radiologist with 10 years of experience. SWU was acquired using a GE LOGIQ E9 (General electric, Boston, MA, USA) machine. The patient fasted for 4 h prior to the study. The examination was performed with the patient lying in the supine or 30-degree lateral decubitus position. The right lobe of the liver was interrogated for the study. The region of interest (ROI) was placed 1.5 to 2 cm away from the liver surface. Care was taken to ensure that the ROI was free of vessels or space-occupying lesions (Figure 1). Five measurements were taken for each subject. The median liver stiffness and inter-quartile range (IQR) of the subject were calculated by the machine. The ratio of IQR/median not exceeding 30% was indicative of an optimal study. MRE was performed on a GE 1.5T Signa HDxt MRI machine (General Electric, Chicago, IL, USA) using a passive driver from Resoundant (Rochester, MN, USA) During the MRE examination, mechanical shear waves were delivered to a fasting patient via an acoustic driver, and its propagation through the liver was imaged with a motion encode gradient (MEG) sequence. An inversion algorithm was used to process the acquired data from the wave images and generate elastograms. Four axial elastogram images were obtained at the largest cross-section of the liver. In this study, regions of interest (ROIs) were manually drawn over the liver. The ROIs were drawn over the magnitude images of the MRE study, taking care to avoid vessels more than 3 mm in diameter and space-occupying lesions in the liver (Figure 2). The ROIs were then copy–pasted onto the grey-scale elastograms which provided the liver stiffness values in kilopascals. ‘Hotspot’ artefacts due to wave interference were also excluded in ROI placement. ROIs were drawn as large as possible in order to include a maximum amount of liver parenchymal in the assessment. For this study, the right liver lobe stiffness was calculated. Cantlie’s line was used to denote the border between the right and left lobes of the liver, using the middle hepatic vein and gallbladder fossa as landmarks. The stiffness of the spleen was also obtained in this study.

### 2.3. Laboratory Parameters

The baseline characteristics of all the patients were recorded. LFTs (liver function tests), including ALT (alanine aminotransferase), AST (aspartate aminotransferase), GGT (gamma-glutamyltransferase), ALP (alkaline phosphatase), GGT (gamma-glutamyl transferase), FBC (full blood count), prothrombin time, international normalized ratio, serum lipid profile, including HDL (high-density lipoprotein), LDL (low-density lipoprotein), and TG (triglycerides), fasting plasma glucose, glycated hemoglobin (HbA1c), vitamin D, thyroid function tests, as well as anthropometric data, were obtained.

All of the above data were obtained within 4 weeks of liver biopsy.

Biochemical NITs were calculated using the established formulae (see Supplementary Index).

The imaging and biochemical NIT cut-off values for advanced fibrosis that were used in this study are based on existing established cut- offs (see Supplementary Index) [8,25,26,27,28,29,30].

### 2.4. Grading of Liver Histology

Liver specimens were obtained either percutaneously or intraoperatively. These were performed either through fine needle trucut biopsy (using a 16-gauge needle) or a wedge biopsy to obtain a minimum 1 cm length of non-fragmented specimen. The wedge biopsies were performed from the left hemiliver in patients undergoing laparoscopic cholecystectomy after the gallbladder specimen was placed in a retrieval bag. The biopsies were performed with scissors, and hemostasis was achieved with electrocautery. The liver specimen was added in the same retrieval bag and extracted via the umbilical port. The specimens were read by an independent, experienced, dedicated histopathologist who was blinded to all clinical data. The liver biopsy specimens were graded according to the criteria of the Non-Alcoholic Steatohepatitis Clinical Research Network (NASH CRN) [31].

Fibrosis was graded as F0 (absent), F1 (mild fibrosis), F2 (significant fibrosis), F3 (advanced fibrosis), or F4 (cirrhosis). The NAFLD non-alcoholic steatohepatitis activity score (NAS) was calculated and reported from 0 to 8 as a sum of the scores for steatosis (0–3), lobular inflammation (0–3), and hepatocellular ballooning (0–2). Advanced fibrosis is defined as patients with fibrosis of grade F3 or higher.

This study was supported by our institutional seed fund program, which comes with a 1-year validity period. Our target sample size was 20 eligible patients, which was based on feasibility in recruitment from our clinic within the study period.

### 2.5. Statistical Analysis

Distribution of the patients’ characteristics is summarized using appropriate descriptive statistics. The distributions of biomarkers and imaging markers are compared between patients with advanced and non-advanced fibrosis using univariate tests. Continuous variables were compared using a t-test, and categorical variables were compared using a chi-squared test. The performance in diagnosing advanced fibrosis for the various NITs studied was assessed by constructing 2 × 2 tables using their established thresholds against the presence or absence of advanced fibrosis determined by the biopsy results. The sensitivity, specificity, positive and negative predictive values, and accuracy of these cut-off points are reported. All statistical tests were performed at a two-sided 5% significance level. A *p* value < 0.05 was used to infer statistical significance.

## 3. Results

### 3.1. Baseline Characteristics

A total of 17 patients were recruited, but 1 patient decided to withdraw from the study as he did not wish to undergo a liver biopsy. Sixteen patients, with an average age of 49.2 years {SD (standard deviation) 11.9 years}, were included in the analysis. Among them, 11 were women (68.7%) and 5 were men (31.3%). In total, 12 patients (75%) were hypertensive, and 11 patients (68.7%) had diabetes. The average BMI was 29.6 kg/m^2^ (SD 3.7 kg/m^2^), and 7 patients (43.7%) had BMI > 30 kg/m^2^. The average waist circumference was 101 cm (SD 9.7 cm) (Table 1).

The biopsy results suggested that nine patients (56.3%) had significant fibrosis (F2 or higher), and six of these patients had advanced fibrosis (F3 or higher). F4 fibrosis was present in one patient (6.3%).

### 3.2. Correlation of Biochemical NITs with Histology for Diagnosis of Advanced Fibrosis

The mean APRI was higher in advanced fibrosis. However, this difference was not statistically significant. A larger proportion of patients with advanced fibrosis also scored higher in the Asia–Pacific NAFLD advanced fibrosis risk assessment, but these differences were not statistically significant either. The distribution of the FIB4 index and AAR was similar between the two groups. While we found that a total of 40% of the patients with non-advanced fibrosis scored ≥2 in the BARD score and were thus incorrectly identified, this proportion was significantly higher in patients with advanced fibrosis (83.4%, *p* value 0.045). Compared to non-advanced fibrosis patients, a higher proportion of patients with advanced fibrosis scored ≥3 in the Asia–Pacific NAFLD advanced fibrosis risk assessment (50% vs. 20%), but this difference did not reach statistical significance. Average liver stiffness was significantly higher in patients with advanced fibrosis, regardless of which method was employed to measure it, compared to patients without advanced fibrosis (Table 2).

### 3.3. Diagnostic Performance of Established Cut-Offs for Imaging and Biochemical NITs

The sensitivity, specificity, positive (PPVs) and negative predictive values (NPVs), and accuracy of the established cut-off points are reported in Table 3. The APRI, BARD score, and Asia–Pacific NAFLD advanced fibrosis risk score have similar levels of accuracy (68.7%), with the APRI having the highest specificity (90%) and BARD score having the highest sensitivity (83.3%) along with NPVs (85.7%) in their respective established cut-offs. Among the imaging markers, the diagnostic accuracy was highest for liver stiffness measured by TE (87.5%), followed by that of MRE (81.2%) and SWU (75%) (Table 3). The established threshold of 8.5 kPa of the TE measurement misclassified two patients, one being a case of a false negative. However, the false negative rate for TE was the lowest (16.7%) among all three imaging markers (33.3% for MRE and 66.7% for SWU).

## 4. Discussion

With the burgeoning global epidemic of NAFLD, especially in the Asian subcontinent, this disease is the most common cause of chronic liver disease worldwide, surpassing viral hepatitis [32].

Fibrosis identification remains one of the most crucial steps in the management of this disease, in addition to lifestyle modifications and risk factor control. There has been a lot of research conducted and interest taken in the best modalities for the detection of non-invasive fibrosis as practitioners move away from liver biopsies in routine clinical practice. This has been gaining much traction in Asia as well.

The FIB4 index is one of the most studied and well-performing NITs for the detection of advanced fibrosis in Asian patients [26,27,28,29]. Sumida and colleagues [26] validated the use of the FIB4 index in 576 Japanese patients with biopsy-proven NAFLD patients and found that when using a cut-off of <1.45, advanced fibrosis could be excluded with 98% certainty. This index performed better than the others studied (NFS, BARD, AAR, APRI), and implementing the FIB4 index could avoid 58% of liver biopsies. Of note, in this study, 73% of the patients were obese as defined by BMI > 25. However, in our study population, FIB4 did not predict or exclude advanced fibrosis accurately. The maximum observed value of FIB4 in our data was 3.0, which is lower than the established cut-off for the detection of advanced fibrosis. Hence, the cut-off of 3.25 misclassified 10 patients without advanced fibrosis as false positives (to have advanced fibrosis), leading to a specificity of 0.

It is interesting to note that in our study, the best-performing marker, the BARD score, which has an 85.7% NPV for the diagnosis of advanced fibrosis, was found to be less predictive of advanced fibrosis in Japanese patients compared to Western patients [25]. The authors postulated that the BARD score was less predictive of advanced fibrosis in the Japanese population due to a lower mean BMI compared to Western patients. In this study, less than 50% of the patients had a BMI of >25, whereas our study included only obese patients with a BMI > 25, suggesting that BARD may be a potentially accurate NIT in obese Asian patients.

The Asia–Pacific NAFLD advanced fibrosis score [8] is a unique score developed for the prediction of advanced fibrosis in Asian patients, where the absence of all three factors (see Supplementary Index) had a predictive value of 95–96% for the absence of advanced fibrosis. We could not find an acceptable level of accuracy in our limited data. Using a score of >3 to diagnose advanced fibrosis, we identified only half of positive patients.

Transient elastography (TE) allows for a non-invasive assessment of liver stiffness by the generation of a low-amplitude mechanical pulse which creates a shear wave. The velocity of this wave is directly related to the stiffness of the liver, which is recorded in kPa. Two probes are available for the measurement of liver stiffness, the standard M probe and XL probe (used for patients for whom the M probe cannot obtain accurate measurements for, the use of which is usually machine-prompted).

Several studies and meta-analysis have reported on the good diagnostic accuracy of TE for predicting advanced fibrosis in NAFLD patients [12,13,30,33,34]. However, as described earlier, Asian data on this are lacking. In the largest systemic review published to date [34], the sAUC (summary area under the curve) for the diagnosis of advanced fibrosis was 0.85 for TE. However, of the 14,609 patients studied, only 38% were from Asian studies. A similar high AUROC (0.85) for TE was reported in another study [30]. However, again, Asians were underrepresented in the patient population (40%). Hence, it is encouraging that our small dataset has similarly shown that TE is the best-performing NIT for the detection of advanced fibrosis. Using a cut-off of >8.5 kPa, TE had a specificity of 90%, with the highest diagnostic accuracy of 87.5% (95% confidence interval 68.7–100%) among all the tested marker panels for the prediction of advanced fibrosis.

It is of interest to note that two earlier studies [12,13] have demonstrated that the failure of the M probe in obtaining an accurate reading was greatest in patients with a BMI >30 kg/m^2^, hence recommending the use of the XL probe instead. However, the authors in this review [30] did not find any significant difference in the diagnostic performance between the two probes in a sensitivity analysis of patients matched to BMI. In our study, we needed to use the XL probe in the five patients with a BMI > 30 as readings with the M probe failed. Given the heterogeneity in patient ethnicities in the existing published studies, more studies are needed to evaluate the accuracy of the M probe and XL probe in Asian patients stratified according to BMI.

A recent study by Tamaki et al. [35], in which more than 60% of the 806 patients studied were of Asian ethnicity, with a median BMI of 28.9, found that MRE had an AUROC of 0.867 in the validation cohort of patients for the detection of advanced fibrosis in patients with NAFLD, mirroring the results of earlier studies, reporting high diagnostic accuracies for MRE in the detection of advanced fibrosis [21,34]. This is the only study to date, correlating with biopsy-proven NAFLD, to include obese Asian patients, and the results are encouraging for MRE to be used as an accurate NIT for the detection of advanced fibrosis in these patients. Our data support the literature that liver stiffness measured using MRE has a good discriminatory ability. It indeed identified cases with high accuracy (81.2%), but it could not pick up two out of six patients with advanced fibrosis, instead it misclassified them as patients without advanced fibrosis, leading to a false negative rate of 33.3%.

Our study had several limitations, the main being the small sample size of our study population. Also, we had a heterogenous sample of liver biopsies, with some being percutaneous core biopsies and others intraoperative wedge biopsies.

The strength of our study includes it being the first prospective cohort study to evaluate the distribution and diagnostic accuracy of serum and radiological biomarkers between obese Asian biopsy-proven NAFLD patients with and without fibrosis. As described earlier, in a similarly designed Thai study [20], the authors reported the high diagnostic accuracies of TE, SWU, APRI, and FIB4 for detecting F1-4 fibrosis. However, the authors compared the performance of these markers against MRE, the performance accuracy of which was discussed earlier, and this has not been well established in obese Asian patients.

It has been well established that Asians have a different body metabolic composition and higher body fat percentage for the same BMI compared to Western patients [36,37]. They also have higher rates of insulin resistance compared to Western patients across similar BMI [37]. Insulin resistance is one of the cardinal features of metabolic syndrome, which is a risk factor for NAFLD. Hence, a “one size fits all” model for both Asian and Western patients for the detection of at-risk NAFLD patients with fibrosis is not optimal. Hence, we need further studies to validate the performance of imaging-based NITs such as MRE and TE, which have high diagnostic accuracies for detecting advanced fibrosis, in obese Asian patients.

## 5. Conclusions

In conclusion, TE is the best-performing NIT for the detection of advanced fibrosis in obese Asian patients with biopsy-proven NAFLD in our pilot study. MRE has also demonstrated a high diagnostic accuracy, but it has a higher false negative rate. Hence, we need further studies to validate the performance of imaging-based NITs such as MRE and TE in obese Asian patients with NAFLD.

Transient elastography is the best-performing non-invasive marker of liver fibrosis in obese Asian patients with non-alcoholic fatty liver disease in our pilot, cross-sectional study.

## Figures and Tables

**Table 1:** Distribution of demographic and clinical characteristics of recruited patients.

Characteristics	Value
Age in years, mean (SD)	49.2 (11.9)
Female, *n* (%)	11 (68.8)
Smoking, *n* (%)	1 (6.3)
Waist circumference in cm, mean (SD)	101 (9.7)
Ferritin in ng/mL, mean (SD)	255.4 (253.4)
Chinese ethnicity, *n* (%)	11 (68.8)
Weight in kg, mean (SD)	80.6 (14.7)
Height in mt, mean (SD)	1.65 (0.09)
Body mass index, mean (SD)	29.6 (3.7)
Hypertension, *n* (%)	12 (75.0)
Hyperlipidemia, *n* (%)	13 (81.3)
Diabetes, *n* (%)	11 (68.8)
Impaired fasting glycemia, *n* (%)	1 (6.3)
Obstructive sleep apnea, *n* (%)	1 (6.3)
Hypothyroidism, *n* (%)	2 (12.5)
Total cholesterol in mmol/L, mean (SD)	5.3 (1.1)
HDL in mmol/L, mean (SD)	1.3 (0.28)
LDL in mmol/L, mean (SD)	3.2 (0.95)
TG in mmol/L, mean (SD)	1.8 (0.73)
Hemoglobin in g/dL, mean (SD)	13.8 (0.99)
Albumin g/L, mean (SD)	43.6 (2.8)
AST IU/L, mean (SD)	53.8 (24.9)
ALT IU/L, mean (SD)	90.5 (50.6)
GGT IU/L, mean (SD)	68.4 (43.0)

**Table 2:** Distribution of non-invasive tests (NITs) for assessment of advanced fibrosis.

Tests	Non-Advanced Fibrosis (*n* = 10)	Advanced Fibrosis (*n* = 6)	*p* Value *
**Biomarkers**			
AST-to-platelet ratio index (APRI), mean (SD)	0.59 (0.25)	0.95 (0.56)	0.173
Fibrosis 4 index (FIB4 Index), mean (SD)	1.15 (0.39)	1.55 (0.96)	0.358
BARD Score, *n* (%)			0.045
0	3 (30.0)	0	
1	3 (30.0)	1 (16.7)	
2	0	4 (66.7)	
3	1 (10.0)	0	
4	3 (30.0)	1 (16.7)	
AST-to-ALT ratio/AAR, mean (SD)	0.67 (0.21)	0.64 (0.09)	0.640
Asia–Pacific NAFLD advanced fibrosis risk score, *n* (%)			0.298
0	3 (30.0)	0	
1	3 (30.0)	3 (50.0)	
2	2 (20.0)	0	
3	2 (20.0)	3 (50.0)	
**Imaging markers**			
Liver stiffness by SWE, mean (SD), kPa	6.25 (1.04)	8.33 (2.13)	0.063
Liver stiffness by TE, mean (SD), kPa	6.39 (1.03)	15.17 (5.67)	0.012
Liver stiffness by MRE, mean (SD), kPa	2.75 (0.49)	4.51 (1.48)	0.032

* *p* values are obtained from independent sample *t*-tests and chi-squared tests, as appropriate.

**Table 3:** Diagnostic performance of established cut-offs for NITs (non-invasive tests) for assessment of advanced fibrosis; values are in percentages (95% confidence interval).

NITs	Cut-Off Value	Accuracy (95% Confidence Interval)	Sensitivity	Specificity	Positive Predictive Value	Negative Predictive Value
**Biomarkers**						
APRI	1	68.7 (50–87.5)	33.3 (0–66.7)	90 (70–100)	66.7 (0–100)	69.2 (57.1–83.3)
FIB4 Index	3.25	37.5	100	0	37.5	NA
BARD	2	68.7 (43.7–87.5)	83.3 (50–100)	60 (30–90)	55.6 (36.4–83.3)	85.7 (60–100)
AAR	0.8	56.3 (31.2–75)	83.3 (50–100)	40 (10–70)	45.4 (30–62.5)	80 (42.5–100)
Asia–Pacific NAFLD advanced fibrosis risk score	3	68.7 (43.7–87.5)	50 (16.7–83.3)	80 (50–100)	60 (20–100)	72.7 (55.6–90.9)
**Imaging indices**						
SWE	8.9	75 (62.5–87.5)	33.3 (0–66.7)	100 (100–100)	100 (100–100)	71.4 (62.5–83.3)
TE	8.5	87.5 (68.7–100)	83.3 (50–100)	90 (70–100)	83.3 (57.1–100)	90 (72.7–100)
MRE	3.64	81.2 (62.5–100)	66.7 (33.3–100)	90 (70–100)	80 (50–100)	81.8 (66.7–100)

## Data Availability

All data generated or analyzed during this study are included in this published article [and its Supplementary Information Files].

## Supplementary Materials

The following supporting information can be downloaded at: https://www.mdpi.com/article/10.3390/medicina60010169/s1. Transient elastography in Asian obese patients.
