# Supplementary material for: Transient Elastography Is the Best-Performing Non-Invasive Test of Liver Fibrosis in Obese Asian Patients with Non-Alcoholic Fatty Liver Disease: A Pilot, Cross-Sectional Study
Source: Medicina (Kaunas). 2024 Jan 17;60(1):169. doi: 10.3390/medicina60010169 (PMC10819647; doi:10.3390/medicina60010169)
Supplement: Supplementary file 1 [file medicina-60-00169-s001.zip › medicina-2792917-supplementary.pdf]

Supplementary Index 1

1. FIB4 (Fibrosis 4 Index) = aspartate aminotransferase (AST) (U/L) × age (years)/platelet count (10^9 /L) × alanine aminotransferase (ALT) (U/L) ½ (25)
2. APRI (AST to platelet ratio index): (AST [IU/L]/upper limit of normal AST [U/L]) × 100/platelet count (10^9/L) (25)
3. BARD index Scale 0-4: BMI ≥ 28 kg/m2 = 1 point AST/ALT ≥ 0.8 = 2 points Diabetes = 1 point(21)
4. Asia pacific NAFLD Advanced fibrosis score: Score 0, low risk; score 1-2, moderate risk; score 3-4, high risk(8)

Asia Pacific NAFLD advanced fibrosis score

Risk factor	Criteria	Points
Age	<55years	0
	>=55 years	2
Diabetes Mellitus	Absent	0
	Present	1
Platelet	>=150x10^9/L	0
	<150x10^9/L	1

**NITs(Non invasive tests)**	**Cut-Off Value (for diagnosis of advanced fibrosis)**
**Biomarkers**	
APRI	1
FIB4 Index	3.25
BARD	2
AAR	0.8
Asia–Pacific NAFLD advanced fibrosis risk score	3
**Imaging indices**	
SWE	8.9
TE	8.5
